# The genome sequence of the sallow kitten, *Furcula furcula *(Clerck, 1759)

**DOI:** 10.12688/wellcomeopenres.18112.1

**Published:** 2022-09-12

**Authors:** Douglas Boyes, University of Oxford and Wytham Woods Genome Acquisition Lab, Darwin Tree of Life Barcoding collective, Wellcome Sanger Institute Tree of Life programme, Wellcome Sanger Institute Scientific Operations: DNA Pipelines collective, Tree of Life Core Informatics collective, Brandon Parker, David Plotkin, Akito Y. Kawahara, Darwin Tree of Life Consortium

**Affiliations:** 1 UK Centre for Ecology and Hydrology, Wallingford, Oxfordshire, UK; 2 McGuire Center for Lepidoptera & Biodiversity, University of Florida, Gainesville, Florida, USA

**Keywords:** Furcula furcula, sallow kitten, genome sequence, chromosomal, Lepidoptera

## Abstract

We present a genome assembly from an individual male *Furcula furcula *(the sallow kitten; Arthropoda; Insecta; Lepidoptera; Notodontidae). The genome sequence is 736 megabases in span. The entire assembly (100%) is scaffolded into 29 chromosomal pseudomolecules, with the Z sex chromosome assembled. The complete mitochondrial genome was also assembled and is 17.2 kilobases in length.

## Species taxonomy

Eukaryota; Metazoa; Ecdysozoa; Arthropoda; Hexapoda; Insecta; Pterygota; Neoptera; Endopterygota; Lepidoptera; Glossata; Ditrysia; Noctuoidea; Notodontidae; Stauropinae; *Furcula*; *Furcula furcula* (Clerck, 1759) (NCBI:txid987943).

## Background

The sallow kitten, *Furcula furcula* (Clerck, 1759), is a holarctic moth belonging to the family Notodontidae (prominent moths) that is commonly found throughout Europe, Asia, and North America ( Heath *et al.*, 1983; Miller *et al.*, 2018; Okagaki, 1958). Adults have a wingspan ranging from 30–36 mm, varying slightly between regions of its geographic distribution, and are white to grey in colour with a large grey band on the dorsal wing surface ( Heath *et al.*, 1983). Larvae can grow up to 35 mm and are bright green with a purple or brown marking on the dorsal side and can be identified by their modified anal prolegs that form a forked tail-like appendage, for which the genus derives its name from (“furca” being Latin for fork) ( Heath *et al.*, 1983).

Larvae have been observed to feed on leaves of poplar, willow, birch, and beech trees ( Robinson *et al.*, 2010; Vorbrodt & Müller-Rutz, 1911). Once mature, larvae crawl down the tree trunk and make a hardened cocoon, consisting of silk and wood pulp, in which they pupate ( Heath *et al.*, 1983; Okagaki, 1958). The moth emerges in the summer, between May and September, with 1–3 generations emerging per year ( Heath *et al.*, 1983; Okagaki, 1958; Robinson *et al.*, 2010). The number of generations depends on the climate of the region, with more generations emerging per year in warmer regions ( Okagaki, 1958). Until recently, *F. occidentalis* (Lintner, 1878) was treated as a subspecies of *F. furcula*. Given the global distribution of *F. furcula*, a fully annotated genome will help provide data needed to understand the link between its genotype and its broad larval host breadth and help distinguish this species from similar ones with overlapping distributions.

## Genome sequence report

**Figure 1.**
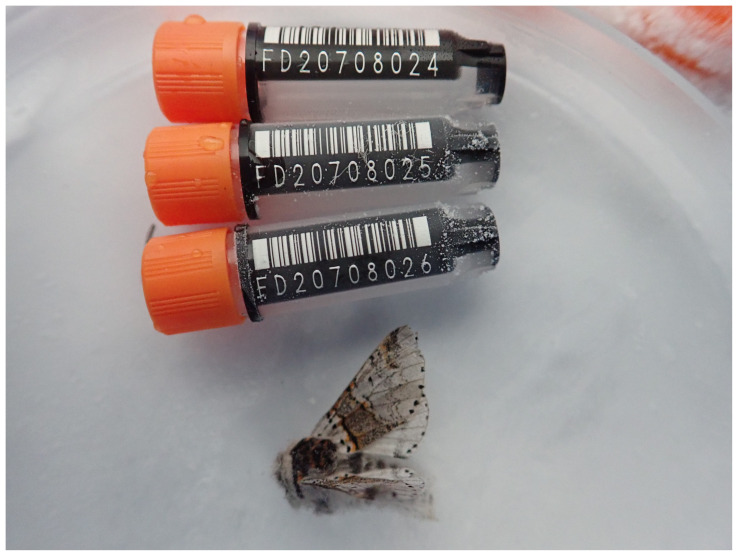
Image of the *Furcula furcula* specimen taken prior to preservation and processing.

The genome was sequenced from a single male *F. furcula* collected from Wytham Woods, Berkshire, UK ( Figure 1). A total of 28-fold coverage in Pacific Biosciences single-molecule HiFi long reads and 62-fold coverage in 10X Genomics read clouds were generated. Primary assembly contigs were scaffolded with chromosome conformation Hi-C data. Manual assembly curation corrected 20 missing/misjoins and removed 5 haplotypic duplications, reducing the assembly size by 0.44% and the scaffold number by 30.43%.

**Figure 2.**
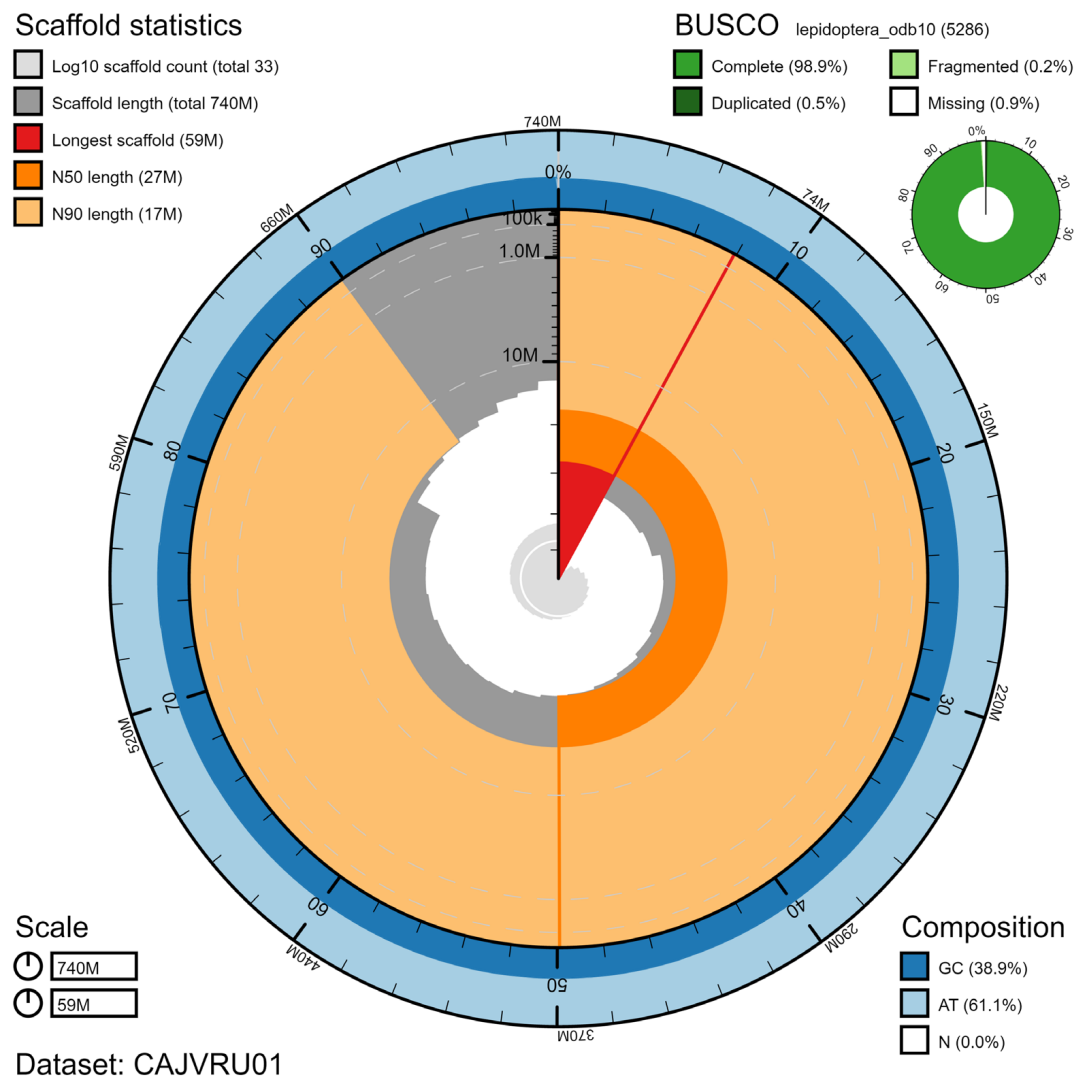
Genome assembly of *Furcula furcula*, ilFurFurc1.1: metrics. The BlobToolKit Snailplot shows N50 metrics and BUSCO gene completeness. The main plot is divided into 1,000 size-ordered bins around the circumference with each bin representing 0.1% of the 736,123,039 bp assembly. The distribution of chromosome lengths is shown in dark grey with the plot radius scaled to the longest chromosome present in the assembly (58,744,975 bp, shown in red). Orange and pale-orange arcs show the N50 and N90 chromosome lengths (27,058,741 and 17,271,334 bp), respectively. The pale grey spiral shows the cumulative chromosome count on a log scale with white scale lines showing successive orders of magnitude. The blue and pale-blue area around the outside of the plot shows the distribution of GC, AT and N percentages in the same bins as the inner plot. A summary of complete, fragmented, duplicated and missing BUSCO genes in the lepidoptera_odb10 set is shown in the top right. An interactive version of this figure is available at https://blobtoolkit.genomehubs.org/view/ilFurFurc1.1/dataset/CAJVRU01/snail.

The final assembly has a total length of 736 Mb in 32 sequence scaffolds with a scaffold N50 of 27.06 Mb ( Table 1). 100% of the assembly sequence was assigned to 29 chromosomal-level scaffolds, representing 28 autosomes (numbered by sequence length) and the Z sex chromosome ( Figure 2– Figure 5; Table 2).

**Table 1.** Genome data for *Furcula furcula*, ilFurFurc1.1.

*Project accession data*	
Assembly identifier	ilFurFurc1.1
Species	*Furcula furcula*
Specimen	ilFurFurc1 (genome assembly, Hi-C, RNA-Seq)
NCBI taxonomy ID	987943
BioProject	PRJEB45669
BioSample ID	SAMEA7746637
Isolate information	Male. Thorax tissue (genome assembly); head tissue (Hi-C); abdomen tissue (RNA-Seq)
*Raw data accessions*	
PacificBiosciences SEQUEL II	ERR6939219
10X Genomics Illumina	ERR6363296-ERR6363299
Hi-C Illumina	ERR6363295
PolyA RNA-Seq Illumina	ERR9434990
*Genome assembly*	
Assembly accession	GCA_911728495.1
*Accession of alternate* *haplotype*	GCA_911728485.2
Span (Mb)	736
Number of contigs	736
Contig N50 length (Mb)	24.9
Number of scaffolds	32
Scaffold N50 length (Mb)	27.06
Longest scaffold (Mb)	32.75
BUSCO * genome score	C:98.9%[S:98.4%,D:0.5%],F:0.2%, M:0.9%,n:5,286

*BUSCO scores based on the lepidoptera_odb10 BUSCO set using v5.3.2. C= complete [S= single copy, D=duplicated], F=fragmented, M=missing, n=number of orthologues in comparison. A full set of BUSCO scores is available at https://blobtoolkit.genomehubs.org/view/ilFurFurc1.1/dataset/CAJVRU01/busco.

**Figure 3.**
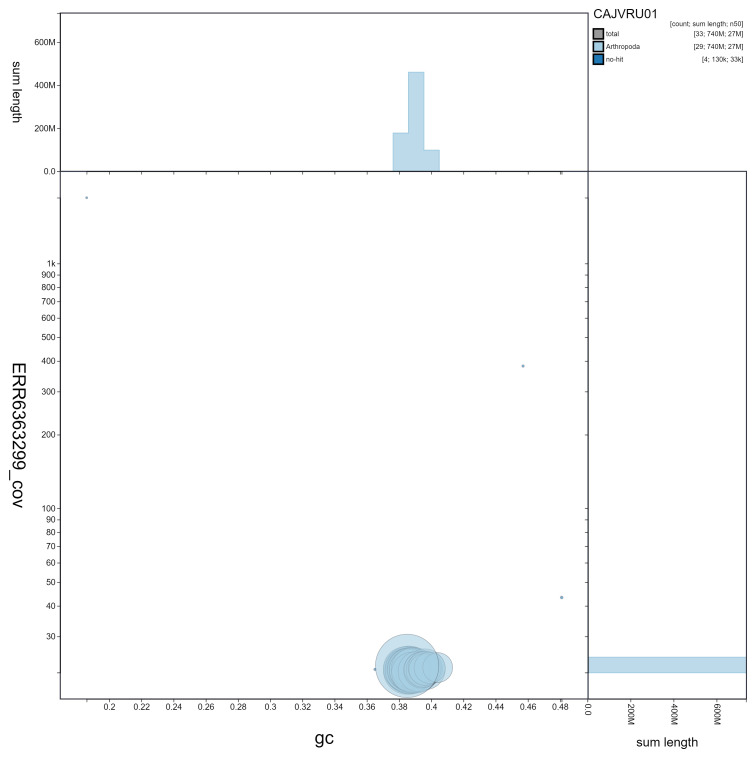
Genome assembly of *Furcula furcula*, ilFurFurc1.1: GC coverage. BlobToolKit GC-coverage plot. Scaffolds are coloured by phylum. Circles are sized in proportion to scaffold length. Histograms show the distribution of scaffold length sum along each axis. An interactive version of this figure is available at https://blobtoolkit.genomehubs.org/view/ilFurFurc1.1/dataset/CAJVRU01/blob.

**Figure 4.**
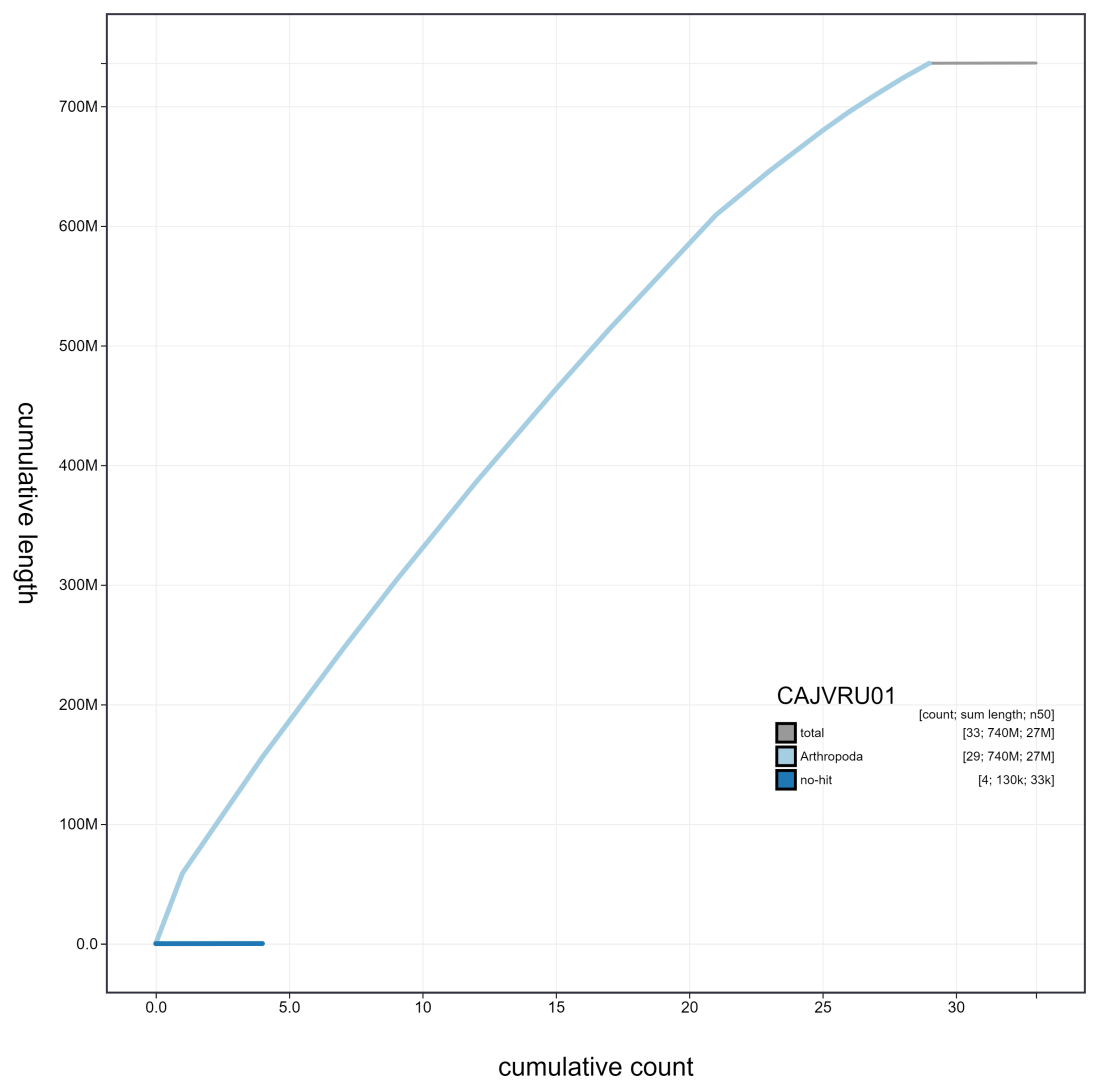
Genome assembly of *Furcula furcula*, ilFurFurc1.1: cumulative sequence. BlobToolKit cumulative sequence plot. The grey line shows cumulative length for all scaffolds. Coloured lines show cumulative lengths of scaffolds assigned to each phylum using the buscogenes taxrule. An interactive version of this figure is available at https://blobtoolkit.genomehubs.org/view/ilFurFurc1.1/dataset/CAJVRU01/cumulative.

**Figure 5.**
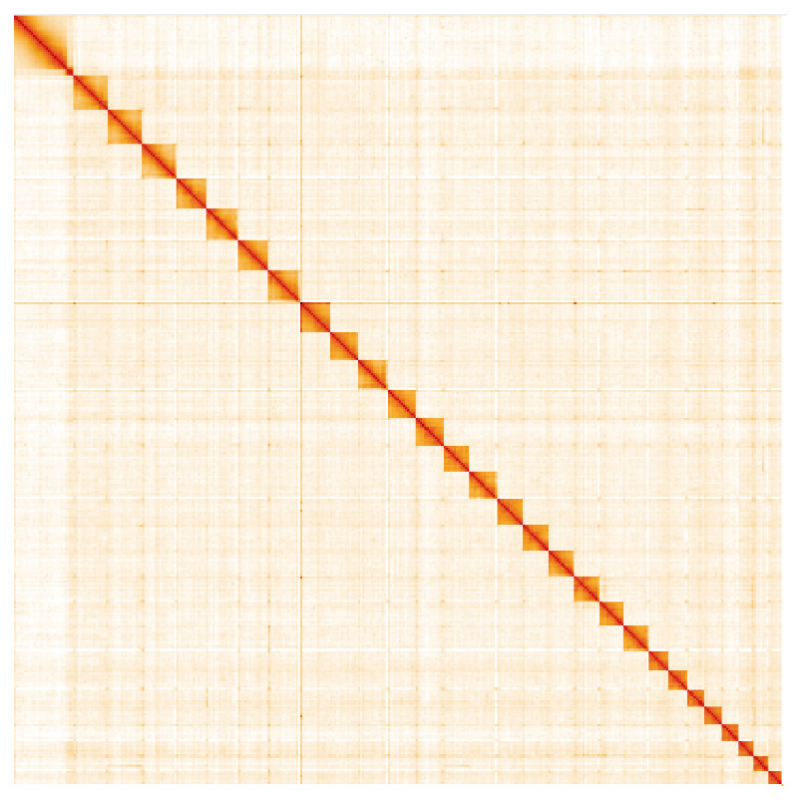
Genome assembly of *Furcula furcula*, ilFurFurc1.1: Hi-C contact map. Hi-C contact map of the ilFurFurc1.1 assembly, visualised in HiGlass. Chromosomes are arranged in size order from left to right and top to bottom. The interactive Hi-C map can be viewed at https://genome-note-higlass.tol.sanger.ac.uk/l/?d=IqL6OXWLRbG3Rb-xprK0Zg.

**Table 2.** Chromosomal pseudomolecules in the genome assembly of *Furcula furcula*, ilFurFurc1.1.

INSDC accession	Chromosome	Size (Mb)	GC%
OU452243.1	1	32.75	38.7
OU452244.1	2	32.66	38.5
OU452245.1	3	31.92	38.6
OU452246.1	4	29.95	38.5
OU452247.1	5	29.86	38.6
OU452248.1	6	29.83	38.6
OU452249.1	7	29.22	38.9
OU452250.1	8	28.36	38.4
OU452251.1	9	27.86	38.5
OU452252.1	10	27.37	38.9
OU452253.1	11	27.06	38.6
OU452254.1	12	26.56	38.7
OU452255.1	13	25.8	38.6
OU452256.1	14	25.6	38.7
OU452257.1	15	25.18	38.7
OU452258.1	16	24.89	39.1
OU452259.1	17	24.26	38.8
OU452260.1	18	23.89	39.6
OU452261.1	19	23.77	38.8
OU452262.1	20	23.58	39.2
OU452263.1	21	18.67	39.4
OU452264.1	22	17.96	39.6
OU452265.1	23	17.27	39.4
OU452266.1	24	16.66	39.5
OU452267.1	25	15.79	39.6
OU452268.1	26	14.31	39.6
OU452269.1	27	13.74	39.9
OU452270.1	28	12.47	40.4
OU452242.1	Z	58.75	38.5
OU452271.1	MT	0.02	19
-	Unplaced	0.11	44.5

The assembly has a BUSCO v5.3.2 ( Manni *et al.*, 2021) completeness of 98.9% (single 98.4%, duplicated 0.5%) using the lepidoptera_odb10 reference set (n=5,286). While not fully phased, the assembly deposited is of one haplotype. Contigs corresponding to the second haplotype have also been deposited.

## Methods

### Sample acquisition and nucleic acid extraction

A single male *F. furcula* specimen (ilFurFurc1) was collected using a light trap from Wytham Woods, Berkshire, UK (latitude 51.772, longitude -1.338) by Douglas Boyes (University of Oxford). The specimen was identified by Douglas Boyes and snap-frozen on dry ice.

DNA was extracted at the Tree of Life laboratory, Wellcome Sanger Institute. The ilFurFurc1 sample was weighed and dissected on dry ice with head tissue set aside for Hi-C sequencing. Thorax tissue was cryogenically disrupted to a fine powder using a Covaris cryoPREP Automated Dry Pulveriser, receiving multiple impacts. Fragment size analysis of 0.01–0.5 ng of DNA was then performed using an Agilent FemtoPulse. High molecular weight (HMW) DNA was extracted using the Qiagen MagAttract HMW DNA extraction kit. Low molecular weight DNA was removed from a 200-ng aliquot of extracted DNA using 0.8X AMpure XP purification kit prior to 10X Chromium sequencing; a minimum of 50 ng DNA was submitted for 10X sequencing. HMW DNA was sheared into an average fragment size between 12–20 kb in a Megaruptor 3 system with speed setting 30. Sheared DNA was purified by solid-phase reversible immobilisation using AMPure PB beads with a 1.8X ratio of beads to sample to remove the shorter fragments and concentrate the DNA sample. The concentration of the sheared and purified DNA was assessed using a Nanodrop spectrophotometer and Qubit Fluorometer and Qubit dsDNA High Sensitivity Assay kit. Fragment size distribution was evaluated by running the sample on the FemtoPulse system.

RNA was extracted from abdomen tissue of ilFurFurc1 in the Tree of Life Laboratory at the WSI using TRIzol, according to the manufacturer’s instructions. RNA was then eluted in 50 μl RNAse-free water and its concentration RNA assessed using a Nanodrop spectrophotometer and Qubit Fluorometer using the Qubit RNA Broad-Range (BR) Assay kit. Analysis of the integrity of the RNA was done using Agilent RNA 6000 Pico Kit and Eukaryotic Total RNA assay.

### Sequencing

Pacific Biosciences HiFi circular consensus and 10X Genomics Chromium read cloud sequencing libraries were constructed according to the manufacturers’ instructions. Sequencing was performed by the Scientific Operations core at the Wellcome Sanger Institute on Pacific Biosciences SEQUEL II (HiFi), Illumina NovaSeq 6000 (10X) and Illumina HiSeq 4000 (RNA-Seq) instruments. Hi-C data were generated in the Tree of Life laboratory from head tissue of ilFurFurc1 using the Arima v2 kit and sequenced on a NovaSeq 6000 instrument.

### Genome assembly

Assembly was carried out with Hifiasm ( Cheng *et al.*, 2021); haplotypic duplication was identified and removed with purge_dups ( Guan *et al.*, 2020). One round of polishing was performed by aligning 10X Genomics read data to the assembly with longranger align, calling variants with freebayes ( Garrison & Marth, 2012). The assembly was then scaffolded with Hi-C data ( Rao *et al.*, 2014) using SALSA2 ( Ghurye *et al.*, 2019). The assembly was checked for contamination as described previously ( Howe *et al.*, 2021). Manual curation was performed using HiGlass ( Kerpedjiev *et al.*, 2018) and Pretext. The mitochondrial genome was assembled using MitoHiFi ( Uliano-Silva *et al.*, 2021), which performs annotation using MitoFinder ( Allio *et al.*, 2020). The genome was analysed and BUSCO scores generated within the BlobToolKit environment ( Challis *et al.*, 2020). Table 3 contains a list of all software tool versions used, where appropriate.

**Table 3.** Software tools used.

Software tool	Version	Source
Hifiasm	0.15.1	Cheng *et al.*, 2021
purge_dups	1.2.3	Guan *et al.*, 2020
SALSA2	2.2	Ghurye *et al.*, 2019
longranger align	2.2.2	https://support.10xgenomics.com/ genome-exome/software/pipelines/ latest/advanced/other-pipelines
freebayes	1.3.1-17- gaa2ace8	Garrison & Marth, 2012
MitoHiFi	2.0	Uliano-Silva *et al.*, 2021
HiGlass	1.11.6	Kerpedjiev *et al.*, 2018
PretextView	0.2.x	https://github.com/wtsi-hpag/ PretextView
BlobToolKit	3.2.6	Challis *et al.*, 2020

### Ethics/compliance issues

The materials that have contributed to this genome note have been supplied by a Darwin Tree of Life Partner. The submission of materials by a Darwin Tree of Life Partner is subject to the Darwin Tree of Life Project Sampling Code of Practice. By agreeing with and signing up to the Sampling Code of Practice, the Darwin Tree of Life Partner agrees they will meet the legal and ethical requirements and standards set out within this document in respect of all samples acquired for, and supplied to, the Darwin Tree of Life Project. Each transfer of samples is further undertaken according to a Research Collaboration Agreement or Material Transfer Agreement entered into by the Darwin Tree of Life Partner, Genome Research Limited (operating as the Wellcome Sanger Institute), and in some circumstances other Darwin Tree of Life collaborators.

## Data availability

European Nucleotide Archive: Furcula furcula (sallow kitten). Accession number PRJEB45669; https://identifiers.org/ena.embl/PRJEB45669.

The genome sequence is released openly for reuse. The *F. furcula* genome sequencing initiative is part of the Darwin Tree of Life (DToL) project. All raw sequence data and the assembly have been deposited in INSDC databases. The genome will be annotated using the RNA-Seq data and presented through the Ensembl pipeline at the European Bioinformatics Institute. Raw data and assembly accession identifiers are reported in Table 1.

## Author information

Members of the University of Oxford and Wytham Woods Genome Acquisition Lab are listed here: https://doi.org/10.5281/zenodo.6418202.

Members of the Darwin Tree of Life Barcoding collective are listed here: https://doi.org/10.5281/zenodo.6418156.

Members of the Wellcome Sanger Institute Tree of Life programme are listed here: https://doi.org/10.5281/zenodo.6866293.

Members of Wellcome Sanger Institute Scientific Operations: DNA Pipelines collective are listed here: https://doi.org/10.5281/zenodo.5746904.

Members of the Tree of Life Core Informatics collective are listed here: https://doi.org/10.5281/zenodo.6125046.

Members of the Darwin Tree of Life Consortium are listed here: https://doi.org/10.5281/zenodo.6418363.
