# Supplementary material for: The genome sequence of the sallow kitten, Furcula furcula (Clerck, 1759)
Source: Wellcome Open Res. 2022 Sep 12;7:229. [Version 1] doi: 10.12688/wellcomeopenres.18112.1 (PMC9984736; doi:10.12688/wellcomeopenres.18112.1)
Supplement: Peer review history

# Peer review: Reviewer response for version 1

**DOI:** 10.21956/wellcomeopenres.20083.r55051

**Published:** 2023-03-03

**Authors:** Danilo T Amaral

**Affiliations:** 1 Graduate School of Biotechnology and Environmental Monitoring (UFSCar),, Sorocaba,, Brazil

I was pleased to review the date note by Boyes et al. entitled “The genome sequence of the sallow kitten, Furcula furcula (Clerck, 1759)”. Genomic information, with high-quality sequencing and assembly, from different species groups, has great importance nowadays for distinct fields, such as evolution, phylogeography, biotechnology, and so forth. Even more, those that present chromosome quality assembly, such as here. The manuscript methodology and results were well-written and detailed.

 As two minor considerations/comments, despite all figures and tables being quite essential to demonstrate the assembly quality, I suggest including figures 3 to 5 and table 3 as supplementary material. Based on the sequencing and assembly methodologies. I would like to know if any other study performed the karyotype of this species (or close species) to determine the number of chromosomes. If so, you may add this information to the text and briefly discuss it.

Are sufficient details of methods and materials provided to allow replication by others?

Yes

Is the rationale for creating the dataset(s) clearly described?

Yes

Are the datasets clearly presented in a useable and accessible format?

Yes

Are the protocols appropriate and is the work technically sound?

Yes

Reviewer Expertise:

Genomics, bioinformatics

I confirm that I have read this submission and believe that I have an appropriate level of expertise to confirm that it is of an acceptable scientific standard.

# Peer review: Reviewer response for version 1

**DOI:** 10.21956/wellcomeopenres.20083.r54246

**Published:** 2023-02-06

**Authors:** Sivasankaran Kuppusamy

**Affiliations:** 1 Loyola College, chennai, India

I appreciate the author for assembling the whole genome sequence of sallow kitten, *Furcula furcula*. The standard methods for sequencing the whole genome, genome assembling technique and software were followed properly.  

**The comments on the manuscripts:**

 Total lengths of raw reads (paired) were not mentioned in the text.

 How many nuclear genes received in the whole genome sequence assembly? The nuclear gene numbers haven’t mentioned in the text.

 The maximum and minimum contig length was not indicated. Total contigs length was not indicated.

 Gene recovery statistics can be given in the text.

Are sufficient details of methods and materials provided to allow replication by others?

Yes

Is the rationale for creating the dataset(s) clearly described?

Yes

Are the datasets clearly presented in a useable and accessible format?

Yes

Are the protocols appropriate and is the work technically sound?

Yes

Reviewer Expertise:

Taxonomy and Phylogenetic analysis of superfamily  Noctuoidea moths

I confirm that I have read this submission and believe that I have an appropriate level of expertise to confirm that it is of an acceptable scientific standard.
